# P16 and HPV Genotype Significance in HPV-Associated Cervical Cancer—A Large Cohort of Two Tertiary Referral Centers

**DOI:** 10.3390/ijms22052294

**Published:** 2021-02-25

**Authors:** Sara da Mata, Joana Ferreira, Inmaculada Nicolás, Susana Esteves, Gonçalo Esteves, Sofia Lérias, Fernanda Silva, Adela Saco, Daniela Cochicho, Mário Cunha, Marta del Pino, Jaume Ordi, Ana Félix

**Affiliations:** 1 Department of Pathology, Instituto Português de Oncologia de Lisboa Francisco Gentil, 1099-023 Lisbon, Portugal; smata@ipolisboa.min-saude.pt (S.d.M.); jferreira@ipolisboa.min-saude.pt (J.F.); slerias@ipolisboa.min-saude.pt (S.L.); 2 Nova Medical School, Universidade Nova de Lisboa, 1169-056 Lisbon, Portugal; fernanda.silva@nms.unl.pt; 3 Institute Clinic of Gynecology, Obstetrics, and Neonatology, Hospital Clínic—Institut d’Investigacions Biomèdiques August Pi i Sunyer (IDIBAPS), University of Barcelona, 08036 Barcelona, Spain; inicolas@clinic.cat (I.N.); mdelpino@clinic.cat (M.d.P.); 4 Clinical Investigation Department, Instituto Português de Oncologia de Lisboa Francisco Gentil, 1099-023 Lisbon, Portugal; sesteves@ipolisboa.min-saude.pt; 5 Department of Pathology, Centro Hospitalar e Universitário de Lisboa Central, 1150-199 Lisboa, Portugal; goncalo.esteves@chlc.min-saude.pt; 6 Department of Pathology, Hospital Clínic, University of Barcelona, 08036 Barcelona, Spain; masaco@clinic.cat (A.S.); jordi@clinic.cat (J.O.); 7 Department of Virology, Instituto Português de Oncologia de Lisboa Francisco Gentil, 1099-023 Lisbon, Portugal; dcochicho@ipolisboa.min-saude.pt (D.C.); mcunha@ipolisboa.min-saude.pt (M.C.); 8 Institut de Salut Global de Barcelona (ISGlobal), 08036 Barcelona, Spain

**Keywords:** cervical cancer, HPV, p16 expression

## Abstract

The expression of p16 is a good surrogate of human papillomavirus (HPV) infection in HPV-associated cancers. The significance of p16 expression, HPV genotype and genera in the outcome of patients with HPV-associated cervical cancer (CC) is unclear. Our aim is to ascertain the prognostic significance of these factors. Data from 348 patients (median age: 47.5 years old) with CC, diagnosed in two referral centers, were retrospectively collected. Advanced disease (FIGO2018 IB2-IV) was present in 68% of patients. A single HPV genotype was identified in 82.8% of patients. The most common HPVs were HPV16 (69%) and HPV18 (14%). HPV genera reflected this distribution. HPV16 tumors presented at an earlier stage. P16 was negative in 18 cases (5.2%), 83.3% of which were squamous cell carcinomas. These cases occurred in older patients who tended to have advanced disease. In the univariate analysis, HPV16 (HR: 0.58; *p* = 0.0198), α-9 genera (HR: 0.37; *p* = 0.0106) and p16 overexpression (HR: 0.54; *p* = 0.032) were associated with better survival. HPV16 (HR: 0.63; *p* = 0.0174) and α-9 genera (HR: 0.57; *p* = 0.0286) were associated with less relapse. In the multivariate analysis, only the International Federation of Gynecology and Obstetrics (FIGO) stage retained an independent prognostic value. HPV16, α-9 genera and p16 overexpression were associated with better survival, although not as independent prognostic factors. Patients with p16-negative HPV-associated CC were older, presented with advanced disease and had worse prognosis.

## 1. Introduction

Human papillomavirus (HPV) is etiologically involved in most cervical cancers (CCs) [1,2,3] and has been identified in approximately 95% of these tumors [4]. HPV is also involved in a subset of tumors of the vagina, vulva, penis, anal canal and head and neck [5]. More than 200 HPV types [6] have been identified, and 20 HPV types are known to cause cervical cancer [7]. Clinically relevant HPV genera such as α-9 and α-7 are the main groups associated not only with invasive CC and its precursor lesion, cervical intraepithelial neoplasia, but also with other cancers associated with this viral infection [8,9]. HPV 16 and 18 are the most prevalent genotypes of each genus, respectively [10,11]. 

Almost all studies, even those using highly sensitive HPV tests, identify a small proportion of CCs negative for HPV [12,13,14]. Remarkably, HPV infection has shown to be of prognostic significance in carcinomas of the vagina, vulva and head and neck, and in all these anatomical locations, HPV-independent tumors have shown an impaired prognosis compared with HPV-associated carcinomas [12,15,16,17,18,19]. This link has also been described in the uterine cervix 12, and the recognition of the biological and clinical relevance of dividing CC into HPV associated and independent was recognized in the last WHO classification [20].

The immunohistochemical overexpression of p16 has been considered to be a good surrogate for HPV infection. Diffuse and strong p16 staining is consistently present in almost all HPV-associated cancers, whereas HPV-independent tumors are typically p16 negative [20,21]. Due to this strong association between p16 overexpression and HPV status, in all the above-mentioned anatomical sites, p16-negative tumors behave more aggressively than p16-positive tumors [22,23,24,25,26]. Interestingly, recent evidence indicates that a small subset of HPV-associated CCs does not overexpress p16 and that these p16-negative HPV-associated CCs behave more aggressively than the conventional HPV-associated CCs overexpressing p16 [27]. However, there is little information on the true frequency and clinical implications associated with the absence of p16 expression in HPV-associated CCs. 

Finally, the prognostic value of the HPV genotype is still unclear, largely because previous studies have yielded conflicting results. Studies on the prognostic value of the most prevalent high-risk HPVs, namely HPV 16 and 18, revealed contradictory positive and negative associations [9,15,28,29]. Discrepancies might be due to differences in sample size, the length of follow-up, assay methods and adjustment for known prognostic factors. Concerning HPV genera, α-9-positive CC has been recently associated with improved local control and poor disease-free survival as well as with distant metastasis-free survival following radiotherapy, as opposed to α-7-positive CC [30,31]. 

This study on HPV genera and genotypes and the p16 prognostic value in HPV-associated CC gathers data from two cohorts of patients, with a long follow-up period of two tertiary referral centers in Portugal and Spain. Partial results from both cohorts have been previously published and include the association between p16 expression and better prognosis [27,32]. Our main aim was to ascertain whether a larger cohort would validate those preliminary results. 

## 2. Results

### 2.1. Patient Clinicopathologic Characteristics 

A total of 348 women with HPV-associated CC were included in this study. The median age at diagnosis was 47.5 years (range: 22–89 years). The most common histological type of CC was squamous cell carcinoma (80%), followed by adenocarcinoma (19%) and four other rarer subtypes (three adenosquamous carcinomas and one neuroendocrine carcinoma). At the time of diagnosis, 68% (*n* = 237) of the patients presented with advanced disease (International Federation of Gynecology and Obstetrics (FIGO) stages 2018 IB2-IV) and 26% (*n* = 92) with lymph node metastases. 

### 2.2. HPV Genotypes, Genera and p16 Expression

The distribution of HPV genotypes and genera among the 348 patients with HPV-associated CC is shown in Table 1, based on the hierarchical approach described by Rad et al. [33]. One or more specific genotypes were identified in 331 CCs, whereas in 17 tumors, it was not possible to identify the specific HPV type(s). Single-type HPV infections were identified in 288 tumors (82.8%) and multiple HPV infections in 43 cancers (12.4%). High-risk and probably high-risk HPV genotypes were detected in 328 (99%) of the 331 tumors where specific HPV types were identified, and only low-risk HPV genotypes were found in 3 carcinomas (1%). HPVs of the α-9 (HPV16, 33, 58, 31, 35 and 52) and α-7 (HPV18, 39, 45, 59 and 68) genera were the most frequently detected. 

HPV16 was identified in 241 (69.0%) tumors (single or multiple infections). The median ages at diagnosis of patients with HPV16 and non-HPV16 tumors were 46 and 49 years, respectively (*p* = 0.08). HPV16 tumors were more likely to present with early disease (36%) compared to non-HPV16 tumors (23%) (*p* = 0.02). The number of patients who presented with lymph node metastases at diagnosis was similar between the HPV16 and non-HPV16 groups (26% vs. 27%, respectively; *p* = 0.85). The association between HPV16 detection and histological type was not significant (squamous cell carcinomas: 80.7% vs. adenocarcinomas: 19.2%; *p* = 0.64). HPV18 was more frequently detected in adenocarcinomas than in squamous cell carcinomas (61.9% vs. 38.1%; *p* < 0.00001).

Of all the 348 tumors, 18 (5%) were p16 negative. The clinicopathological characteristics (age, histological type, FIGO staging, lymph node metastasis) of the p16-positive and p16-negative tumors are shown in Table 2. These p16-negative tumors were more likely to occur in older women (median age: 58.5 vs. 47.0 years, *p* = 0.05) with advanced disease (FIGO IB2-IV 89% vs. 68%, *p* = 0.06). Most p16-negative tumors were squamous cell carcinoma (SCC) (83.3%), and the remaining four cases were ADC (16.7%). Regarding the HPV genotypes of p16-negative tumors, eleven cases (61%) had HPV16 infection. Two p16-negative cases had multiple infections with HPV16, HPV31, HPV16 and HPV33. The four p16-negative adenocarcinomas had single HPV infections. In two cases each, HPV18 and HPV16 were identified. In the remaining four cases of this subgroup (all SCC), two harbored other high-risk HPV genotypes (HPV56 and HPV33), and a probably high-risk genotype (HPV70) and low-risk HPV genotype (HPV6) were found in one case each. Accordingly, the HPV genera of the p16-negative tumors were mainly α-9 (67%), followed by α-7 (17%), non-α-9 non-α-7 (11%) and unknown genera (6%).

No significant statistical differences were found between the p16-positive and p16-negative subgroups regarding the center of origin, histology, presence of lymph node metastases or HPV genotype and genera (Table 1). 

### 2.3. Survival and Prognostic Variables

After a median follow-up of 111.6 months (95% CI: 95.7–130.7 months), 31% (*n* = 109) of the patients had disease relapse, and 22% (*n* = 78) patients died due to disease progression. A total of 35 (10%) patients died due to non-CC-related causes and 6 from unknown causes (2%). The median 5- and 10-year disease-free survival (DFS) rates were 67.8% (95% CI: 62.8–73.2%) and 65.4% (95% CI: 60.2–71.1%), respectively. The median 5- and 10-year disease-specific survival (DSS) rates were 75.9% (95% CI: 71.2–81%) and 74.3% (95% CI: 69.3–79.6%), respectively.

**Figure 1:**
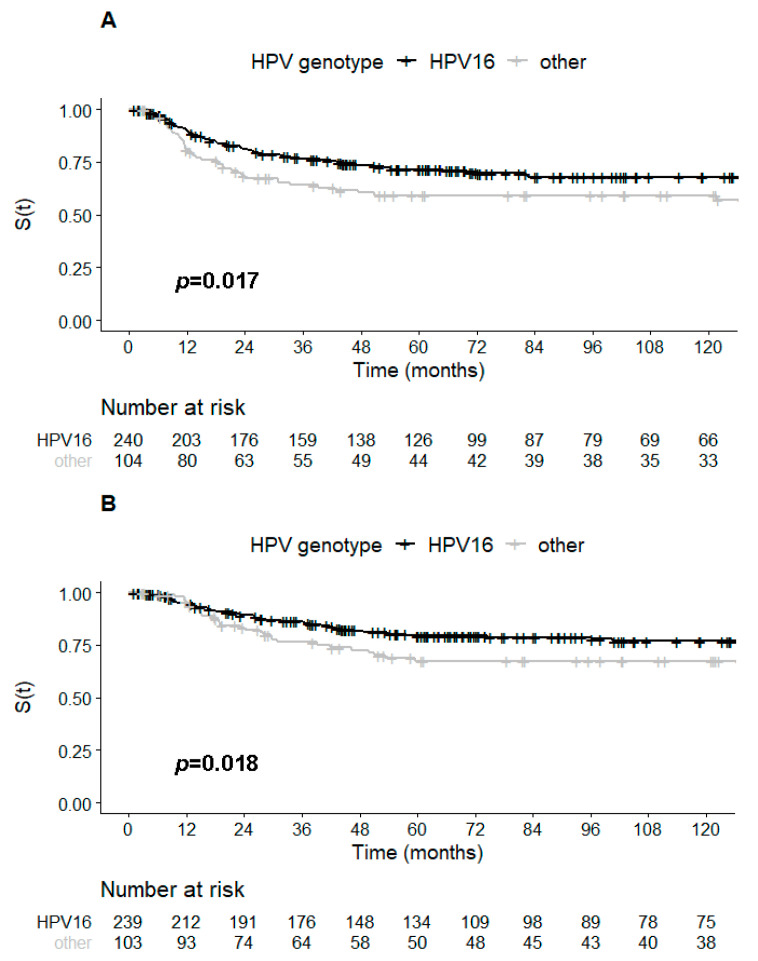
Kaplan–Meier curves of the cervical cancers stratified according to HPV16 status or other HPV: (**A**) disease-free survival; (**B**) disease-specific survival. HPV, human papillomavirus, S(t), probability of survival.

Patients with HPV16-associated tumors had significantly better five-year DFS (71.6% vs. 59.1%, *p* = 0.017) and DSS rates (79.5% vs. 67.5%, *p* = 0.018) than patients with tumors associated with other HPV types (Figure 1A,B). Concerning the histological type of CC, HPV16 detection was associated with better DSS in SCC (*p* = 0.016) but not in ADC (*p* = 0.98).

**Figure 2:**
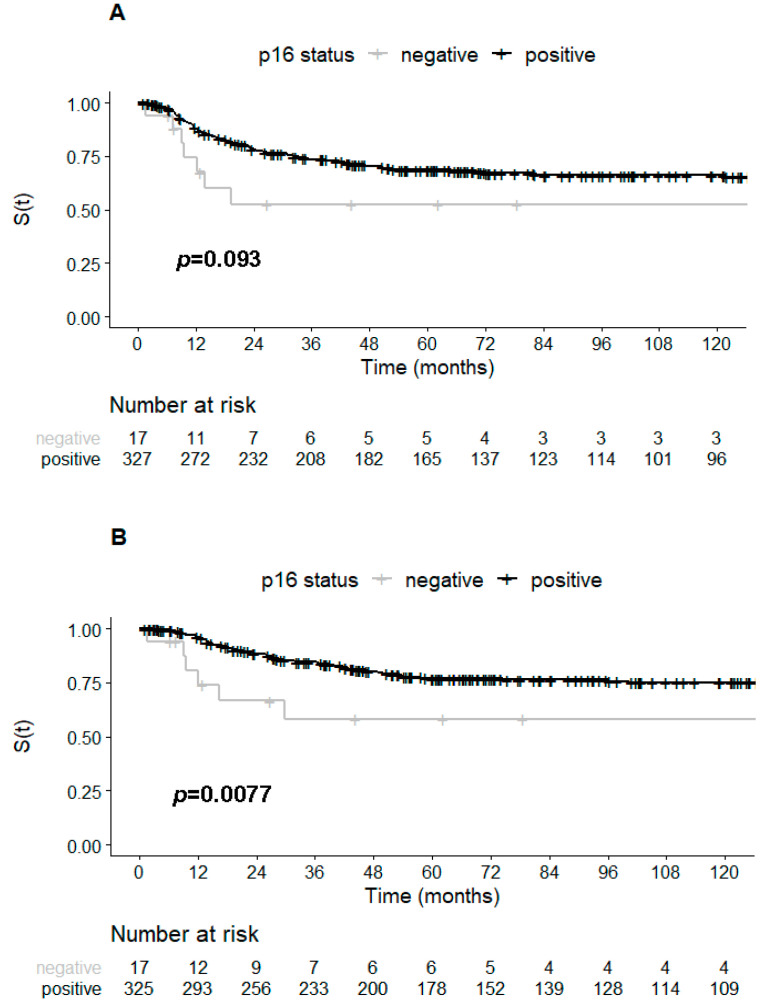
Kaplan–Meier curves of the cervical cancers stratified according to p16 expression (positive/negative): (**A**) disease-free survival; (**B**) disease-specific survival. S(t), probability of survival.

**Figure 3:**
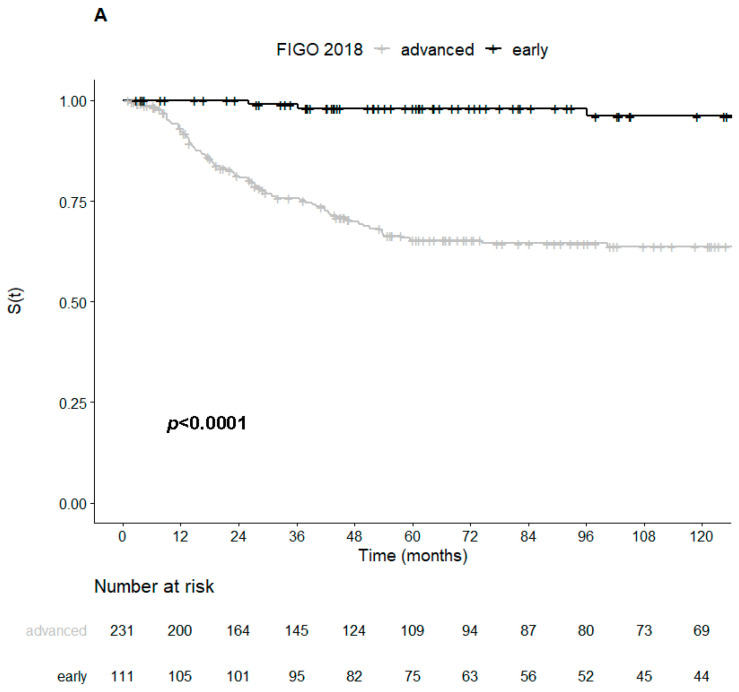

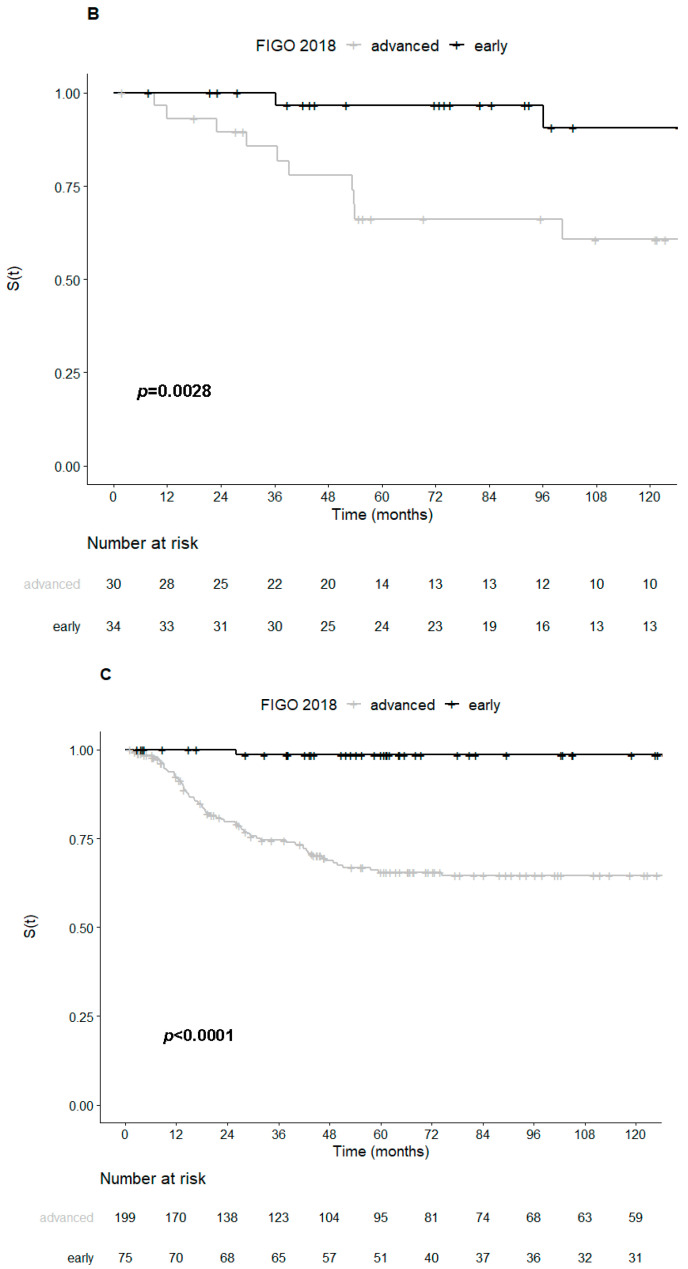
Kaplan–Meier curves for disease-specific survival of patients with cervical cancer stratified according to the FIGO stage (early/advanced): (**A**) all histological types; (**B**) adenocarcinoma; (**C**) squamous cell carcinoma.

Patients with tumors overexpressing p16 also had longer five-year DSS rates than patients with p16-negative tumors (76.8% vs. 58.2%, *p* = 0.0077), but no difference between five-year DFS rates were observed between tumors with and without p16 overexpression (68.6% vs. 52.6%, *p* = 0.093) (Figure 2A,B and Figure 3). 

In the univariate survival analysis, the presence of HPV16 genotype, p16 overexpression and α-9 genera were significantly associated with better survival (Table 3). Tumors with an HPV16 genotype and α-9 genera were also significantly associated with a lower risk of disease progression, but this was not the case for p16 overexpression. As expected, patients with early-stage FIGO tumors were also shown to have a lower risk of both death and relapse. 

On multivariate analysis, only the early FIGO stage remained associated with better survival and a lower risk of disease relapse (Table 4).

## 3. Discussion

In this study, we reviewed 348 cases of HPV-associated invasive CCs with prolonged follow-up (median: 9.25 years) and evaluated HPV genotypes, genera and p16 expression. We adjusted the survival analysis for age at diagnosis and FIGO stage 2018.

As previously reported [10,11], the most frequent HPV genotypes linked to an increased risk of cancer development and the most prevalent in invasive CC were HPV16 (70.5%) and HPV18 (9.7%). HVP18-positive women had a relatively higher proportion of ADC diagnosis than SCC, in keeping with the results of the previously published subcohort and other reports [27,28,34,35]. 

There are contradictory data regarding the significance of HPV genotypes on the clinical outcome of patients with HPV-associated cervical cancer [11,36]. Several studies have suggested a favorable impact of HPV16 on the outcome of patients with CC relative to other high-risk HPV genotypes [9,15,36]. However, the reverse has also been published [28,35]. A trend towards HPV16 and a better prognosis were identified in the results regarding the overall population and both subpopulations from Spain and Portugal used in this study [27,32]. In this larger cohort, HPV16 was associated with a better clinical outcome, although we did not consolidate this finding into a significant result in the multivariate analysis. Even though there was no significant difference in age between patients with HPV16-positive tumors and patients with other HPV types, more patients of the latter group presented with advanced disease. This could explain, at least partially, the difference in the prognostic value observed in the univariate and multivariate analysis.

Clustering HPV genotypes into their genera could be clinically more relevant. HPV α-7-positive CC, which includes HPV18, has been associated with worse local control after radiotherapy, relative to HPV α-9-positive cases, which include HPV16 [30]. In this study, HPV genera α-9 and α-7 did not significantly impact the clinical outcome when adjusted for FIGO stage and age, although there was a positive association between α-9 genera and better outcome in the univariate analysis.

In this cohort, 5% of HPV-associated invasive CC were p16 negative. A variety of mechanisms could explain the negativity of p16 in HPV-associated carcinomas. Loss of heterozygosity has been reported in HPV-associated carcinomas, as have point mutations of CDKN2A or the silencing of the CDKN2A gene by promoter hypermethylation of the promoter [37,38,39,40]. Any of these mechanisms may explain one case in our series, where p16-negative invasive CC was associated with a p16-positive intraepithelial lesion [27]. The true oncogenic value of HPV DNA detection has been debated in other organs, where simple HPV DNA detection is not considered sufficient [41]. In one case of our series, the detected HPV type was HPV6, a known low-risk type that is unlikely related to p16 overexpression and carcinogenesis [42]. Interestingly, the patient in question had an early-stage SCC and was alive and well more than 15 years after the initial diagnosis. 

Inactivation of p16INK4a has been associated with more aggressive behavior in several cancers [37,43,44]. However, little is known about the role of p16 expression in the prognosis of HPV-associated CC. In CC, p16 immunohistochemical overexpression is frequently used as a surrogate for HPV infection, although p16-negative tumors with HPV infection do exist [37,38,45]. A recent meta-analysis reported better DFS of CCs associated with the overexpression of p16 [46], but this study did not stratify patients for HPV DNA detection and was thus blind for HPV status. This is important as HPV-independent CC has been associated with a worse outcome [12]. In the previously published subpopulation of this study, p16-negative cases showed increased mortality, namely worse overall survival [27]. In this larger cohort, we confirmed this trend, but this result was not statistically significant. As the subgroup of patients with p16-negative tumors remains a very small one, this could impair our analysis and thus explain the lack of statistical significance in the multivariate analysis. Interestingly, despite the small number of patients with p16-negative tumors, there was a statistically significant difference in age between this subgroup and the remaining patients who were approximately one decade younger. Patients with tumors negative for p16 also tended to present with more advanced disease. As both these factors have been included in the multivariate survival analysis, they could partially account for the difference in prognosis observed in the univariate analysis [27]. The HPV oncogenes E6 and E7 can inactivate Rb protein and thus lead to p16 overexpression. In a Norwegian study, the prognostic significance of HPV DNA and E6/E7 mRNA, the presence of specific types and the physical state of HPV DNA were explored in 202 cervical squamous cell carcinomas. Absence or non-detectable levels of high-risk (types 16, 18, 31, 33, 35, 45, 52 and 58) E6/E7 mRNA was associated with poor overall survival in both univariate analysis and multivariate analysis [47].

## 4. Materials and Methods

### 4.1. Study Design

This is a retrospective study conducted in two tertiary referral centers (Instituto Português de Oncologia de Lisboa Francisco Gentil E.P.E. (IPOLFG) and the Hospital Clinic of Barcelona (HCB) regarding 348 patients with HPV-associated CC, diagnosed between 1980 and 2016 and 1999 and 2016 in the Lisbon and Barcelona centers, respectively. The Portuguese cohort included all consecutive cases diagnosed during the months of March and April in the pair-years of the 1980, 1990 and 2000 decades. Since 2005, all cases of CC with the inclusion criteria were included. Cases were not selected by organized screening practices. The Spanish cohort included all women with CC admitted to the Gynecological Oncology Unit of the Hospital Clinic of Barcelona from January 1999 to January 2016. The inclusion criteria were: (1) histologically confirmed diagnosis of CC, (2) a paraffin block with an available tumor tissue for HPV DNA genotyping and p16 immunostaining and (3) positive result of the HPV DNA test. Clinical features and follow-up were recorded, and pathological parameters were reviewed. Tumors were restaged (SM/AF and IN) according to the 2018 International Federation of Gynecology and Obstetrics (FIGO) staging system. Lymph node metastasis status was determined either on the histopathologic result or the interpretation of radiologic imaging (computerized tomography/magnetic resonance imaging or positron emission tomography with F18-fluorodeoxyglucose) of pelvic and/or para-aortic lymph nodes. Cases with disease persistence after initial treatment were excluded from this study.

Data collection and analysis were approved by the Ethical Committees of each institution (IPOLFG—UIC/2017/1107 approved on 06/07/2017; HCB - 2015/0517 approved on 03/06/2015). Patient consent was waived due to the retrospective nature of the study and the analysis used anonymous clinical data.

### 4.2. Clinical Management and Treatment

The IPOLFG series collected a large span of patients diagnosed and treated from 1988 to 2016. Patients with early-stage cervical cancer diagnosed before 1998 underwent loop electrosurgical excision or brachytherapy followed by radical hysterectomy and, in selected cases, pelvic and/or para-aortic lymph node dissection. Since 2000, patients with adverse risk factors (positive lymph nodes, positive margins, vascular invasion, parametrium or deep cervical stromal invasion) received adjuvant radiotherapy or chemoradiotherapy. Advanced-stage patients received radiotherapy or chemoradiotherapy. Clinical follow-up was performed every 3 months for 2 years, every 6 months from 2 to 5 years and annually thereafter. The treatment and follow-up strategy for the 190 patients from the HCB cohort has been described in detail elsewhere [27], but a summary is provided below. Women with FIGO 2009 stage IA1 tumors without lymphovascular space invasion underwent a loop electrosurgical excision procedure or extrafascial hysterectomy. Patients with FIGO 2009 stage IA1 with lymphovascular invasion, IA2, IB1 or IIA tumors underwent laparoscopy with intraoperative sentinel lymph node evaluation or pelvic lymphadenectomy. Patients with negative nodes were treated either with radical vaginal hysterectomy assisted by laparoscopy or with radical trachelectomy, while patients with positive nodes underwent complete para-aortic and selective pelvic lymphadenectomy, removing all suspicious or enlarged lymph nodes without hysterectomy. Patients with FIGO 2009 stage IB2, IIB or III cervical cancer underwent a complete para-aortic lymphadenectomy with selective pelvic lymphadenectomy. According to the current guidelines for cervical cancer treatment, patients showing risk factors after radical surgery received adjuvant radiotherapy or chemoradiotherapy. Women with stage IV tumors were treated with chemotherapy or chemoradiotherapy, as described elsewhere. 

### 4.3. Histopathological Evaluation

Tumor samples of formalin-fixed, paraffin-embedded tissue from biopsies or surgical specimens were stained with hematoxylin and eosin and p16 immunohistochemistry. For the IPOLFG cases, tissue microarrays were made, each represented by 3 cores of 1.5 mm in diameter, retrieved from different areas of the tumor. 

The human monoclonal p16 antibody, clone E6H4 (Cat. Number: 805-4713, Roche Tissue Diagnostics, prediluted for 4 min; pretreatment, ULTRA CC1, 64 min; Optiview DAB IHC Detection Kit, Ventana Medical Systems, Tucson, Arizona, USA), was used, following the manufacturer’s protocol (the BenchMark ULTRA IHC/ISH automatic staining platform of Ventana Medical Systems or the Autostainer Link 48 automated system of Dako Co.). Cases with diffuse and strong nuclear and cytoplasmatic staining in all viable tumor cells were considered positive for p16. Cases with irregular or focal staining were considered negative [27].

### 4.4. HPV DNA Detection and Genotyping

DNA extraction was performed in formalin-fixed, paraffin-embedded tissue. Isolated DNA was used for HPV detection and genotyping with SPF10 PCR-DEIA-LiPA25 system (Fujirebio-Gent, Belgium) [48]. This platform allows for the simultaneous detection of 32 HPV types including high-risk (16, 18, 31, 33, 35, 39, 45, 51, 52, 56, 58, 59, and 68), probably high-risk HPV types (26, 53, 66, 70, 73 and 82) and low-risk HPV types (6, 11, 40, 42, 43, 44, 54, 61, 62, 67, 81, 83 and 89). HPV DNA-positive specimens not hybridizing with any of the 32 probes were classified as HPV type X (HPV X or undetermined type).

### 4.5. Statistical Analyses

Disease-free survival (DFS) was defined as the time from diagnosis to first recurrence (histologically proven or clinically/radiologically suspected), regardless of the recurrence site. Disease-specific survival (DSS) was defined as the time from diagnosis to death from disease. Patients without follow-up information concerning disease relapse (*n* = 4), and with an unknown cause of death (*n* = 6) were excluded from the survival analysis. 

For analysis purposes, FIGO staging was grouped in early (stages IA1 to IB1) and advanced (stages IB2 to IV) cervical cancer. Cases with a single HPV type were considered as single HPV infections, whereas cases with two or more HPV genotypes were classified as multiple HPV infections. Cases with HPV X were excluded from the analysis of single vs. multiple infections. For HPV genotype analysis purposes, HPV-16-positive tumors included single or multiple HPV 16 infections, and all remaining genotypes, including HPV X, were considered as non-HPV 16 types. Patients with HPV-associated cancer with an undetermined genotype were considered to have an unknown genus (*n* = 17).

We conducted a descriptive analysis using absolute and relative frequencies for categorical variables and the median, minimum and maximum for age. Pearson’s chi-squared test or Fisher’s exact test, as appropriate, were used to evaluate the associations between p16 status at diagnosis and other categorical clinicopathological baseline variables. Mann–Whitney–Wilcox test was used to evaluate the association between age at diagnosis and p16 status. 

Five- and 10-year DFS and DSS rates were calculated using the Kaplan–Meier method, and the log-rank test was used for survival curve comparison. We fitted Cox models to analyze the prognostic factors p16 status (positive vs. negative), HPV genotype (HPV 16 vs. other) and HPV genera (α-7 vs. α-9 vs. α-7 and α-9 vs. other genera). We conducted univariate and multivariable analyses controlling for the potential confounders FIGO stage and age at diagnosis. Cox model assumptions were checked using the Schoenfeld residuals to test the proportional hazards assumption and the Martingale residuals to check the functional form of the age covariate. We considered a significance level of 5% and did not make any adjustment for multiple comparisons. All analyses were performed with R [49], using the “survival” [50,51] and “survminer” [52] packages.

## 5. Conclusions

In our series, HPV16, α-9 genera and p16 overexpression were associated with better survival in patients with HPV-associated CC, although we could not validate the independent prognostic value of these factors. Our results confirm the existence of a small number of HPV-associated CCs that do not overexpress p16 (5%). These patients tend to be older and present with advanced disease, both of which are poor prognostic factors. As our and previous results reflect the same prognostic trend for p16, the small number of cases negative for p16 could be partially responsible for the lack of statistical significance on multivariate analysis. This subpopulation warrants more studies to be better characterized.

## Figures and Tables

**Table 1:** Distribution of human papillomavirus (HPV) types in patients with HPV-associated cervical cancer.

Variables	*n* (%)
Type of HPV infection (*n* = 348)	
Single	288 (82.8%)
Multiple	43 (12.4%)
Unknown (HPVX)	17 (4.9%)
HPV type	
Single infection (*n* = 288)	
HPV16	203 (70.5%)
HPV18	28 (9.7%)
HPV33	12 (4.2%)
HPV45	8 (2.8%)
HPV35	7 (2.4%)
Others *	30 (10.4%)
Multiple infection (*n* = 43)	
HPV16, HPV18	13 (30.2%)
HPV16, HPV33	9 (20.9%)
Other double infections **	14 (32.6%)
Triple infections ***	6 (14.0%)
Quadruple infection ****	1 (2.3%)
HPV genera (*n* = 348)	
α-9	254 (73.0%)
α-7	48 (13.8%)
α-7 and α-9	21 (6.0%)
Non-α-7/α-9	8 (2.3%)
Unknown (HPVX)	17 (4.9%)

HPV, human papillomavirus; *n*, number. * Single infections with HPV6 (1 case), HPV39 (3 cases), HPV44 (1 case), HPV52 (4 cases), HPV53 (2 cases), HPV56 (2 cases), HPV58 (3 cases), HPV59 (4 cases), HPV66 (1 case), HPV 68 (3 cases), HPV70 (1 case) ** Double infection with HPV16 and 26 (1 case), HPV16 and 31 (2 cases), HPV16 and 45 (2 cases), HPV16 and 51 (1 case), HPV16 and 52 (1 case), HPV16 and 56 (1 case), HPV16 and 59 (1 case), HPV26 and 35 (1 case), HPV33 and 11 (1 case), HPV33 and 66 (1 case), HPV45 and 51 (1 case), HPV52 and 68 (1 case). *** Triple infection with HPV16, 18 and 33 (1 case), HPV16, 18 and 45 (1 case), HPV16, 18 and 58 (1 case), HPV 16, 18 and 53 (1 case), HPV16, 18 and 11 (1 case), HPV16, 18 and 56 (1 case). **** Quadruple infection with HPV16, 18, 31 and 52.

**Table 2:** Clinicopathologic characteristics and p16 status of patients with HPV-associated cervical cancer.

Variables	Overall Sample (*n* = 348)	p16 Positive (*n* = 330)	p16 Negative (*n* = 18)	*p*-Value
Age, years old				
median (min–max)	47.5 (22–89)	47 (22–87)	58.5 (35–89)	0.04
Histology, *n* (%)				
SCC	280 (80%)	266 (81%)	14 (78%)	0.82
ADC	64 (19%)	60 (18%)	4 (22%)	
Other	4 (1%)	4 (1%)	0	
FIGO stage 2018, *n* (%)				
Early (IA1 to IB1)	111 (32%)	109 (33%)	2 (11%)	0.05
Advanced (IB2 to IV)	237 (68%)	221 (67%)	16 (89%)	
Lymph node metastases, *n* (%)				
No	256 (74%)	243 (74%)	13 (72%)	0.89
Yes	92 (26%)	87 (26%)	5 (28%)	
HPV genotype, *n* (%)				
HPV16	241 (69%)	230 (70%)	11 (61%)	0.44
Other	107 (31%)	100 (30%)	6 (39%)	
Center, *n* (%)				
IPOLFG	158 (45%)	148 (45%)	10 (56%)	0.37
HCB	190 (55%)	182 (55%)	8 (44%)	

Min, minimum, max, maximum, *n*, number, SCC, squamous cell carcinoma, ADC, adenocarcinoma, FIGO, International Federation of Gynecology and Obstetrics, HPV, human papillomavirus, IPOLFG, Instituto Português de Oncologia de Lisboa Francisco Gentil E.P.E, HCB, Hospital Clinic Barcelona.

**Table 3:** Univariate Cox model for mortality and relapse.

Variables	Mortality			Relapse		
	HR	95% CI	*p*-Value	HR	95% CI	*p*-Value
HPV genotype						
Other	1			1		
HPV16	0.58	0.37–0.92	**0.0198**	0.63	0.43–0.92	**0.0174**
P16						
Negative	1			1		
Positive	0.37	0.17–0.79	**0.0106**	0.52	0.24–1.13	0.0991
HPV genera						
α-7	1			1		
α-9	0.54	0.31–0.95	**0.032**	0.57	0.35–0.94	**0.0286**
α-7 and α-9	0.77	0.28–2.11	0.612	0.82	0.35–1.93	0.649
Other	0.95	0.28–3.26	0.935	0.71	0.21–2.38	0.5764
FIGO stage 2018						
Early	0.07	0.02–0.22	**<0.001**	0.21	0.11-0.38	**<0.001**
Advanced	1			1		

HR, hazard ratio, CI, confidence interval, HPV, human papillomavirus; FIGO, International Federation of Gynecology and Obstetrics, *p*-value < 0.05 are in bold.

**Table 4:** Multivariate Cox model for mortality and relapse.

Variables	Mortality			Relapse		
	HR	95% CI	*p*-Value	HR	95% CI	*p*-Value
HPV genotype						
Other	1			1		
HPV16	0.71	0.42–1.11	0.141	0.71	0.47–1.08	0.086
P16						
Negative	1			1		
Positive	0.5	0.22–1.10	0.085	0.65	0.30–1.40	0.271
FIGO stage 2018						
Early	0.08	0.02–0.25	**<0.001**	0.21	0.11–0.39	**<0.001**
Advanced	1			1		
Age						
Per additional year	1.01	0.99–1.02	0.209	1.00	0.98–1.01	0.634

HR, hazard ratio, CI, confidence interval, HPV, human papillomavirus; FIGO, International Federation of Gynecology and Obstetrics. *p*-value < 0.05 are in bold.

## Data Availability

The data presented in this study are available on request from the corresponding author. The data are not publicly available due to ethical reasons.
